# Development and validation of a nomogram for predicting ADL outcomes in patients undergoing subacute stroke rehabilitation based on machine learning and standard bedside clinical data: a retrospective cohort study

**DOI:** 10.3389/fneur.2026.1831565

**Published:** 2026-06-19

**Authors:** Xinye Chen, Juming Liu, Jiawei Qin, Xi Qin, Changyu Ju, Suchen Zhao, Qianqian Sun

**Affiliations:** 1 School of Computer Science, Guangdong Polytechnic Normal University, Guangzhou, China; 2 Hubei University of Arts and Science Affiliated Xiangyang Central Hospital, Xiangyang, Hubei, China; 3 Department of Rehabilitation Medicine, Quanzhou First Hospital, Quanzhou, Fujian, China; 4 Rehabilitation Medicine and Rehabilitation Engineering Technology Xiangyang Key Laboratory, Xiangyang, Hubei, China

**Keywords:** activities of daily living, machine learning, predictive model, prognosis, stroke rehabilitation

## Abstract

**Background:**

The subacute phase is a key period for stroke recovery, yet there is a lack of simple and effective indicators to predict rehabilitation outcomes. This study aims to develop and validate a predictive model for assessing patients’ activities of daily living (ADL) recovery at 3 months, providing valuable insights to guide clinical rehabilitation decisions.

**Methods:**

This retrospective cohort study included patients admitted to rehabilitation within 7 to 30 days after their first stroke. Data were obtained from the electronic medical record system. Patients were divided into a training cohort (270 patients, 2021–2022) and a validation cohort (165 patients, 2023–2024). ADL independence was defined by a Barthel Index (BI) score of ≥60. The primary outcome was the ADL status at 3 months after the initiation of rehabilitation. Feature selection was performed using univariate analysis and Elastic Net regression, followed by logistic regression modeling. The optimal model was selected based on its AUC in the validation cohort, ensuring a balance between sensitivity and specificity. The final model was presented as a nomogram.

**Results:**

The 3-month prediction model (ADL-3 M) includes the Braden score, baseline BI score, and age. SHAP analysis revealed that the Braden score was the most significant predictor for the 3-month outcome. The AUC for ADL-3 M was 0.832 (95% CI: 0.779–0.885) in the training cohort and 0.866 (95% CI: 0.806–0.926) in the validation cohort.

**Conclusion:**

The simplified model constructed using routine bedside indicators (age, baseline BI score, and Braden score) effectively predicts the ADL recovery of subacute stroke patients at 3 months post-rehabilitation. This nomogram tool is intuitive and easy to use, providing clinical support for individualized rehabilitation plan development, patient prognosis communication, and resource allocation.

## Introduction

Stroke is the leading cause of disability worldwide, and patients often require long-term post-stroke care and rehabilitation (1, 2). Limitations in activities of daily living (ADL) are among the most severe disabling sequelae following stroke, significantly impacting patients’ independence, community reintegration, and quality of life (3, 4). Therefore, early and accurate prediction of medium-term ADL recovery potential—particularly the likelihood of regaining independence in ADL—is essential for clinical decision-making, rehabilitation planning, and resource allocation (5). Timely forecasts of ADL recovery can also help patients and their families prepare for post-stroke consequences and encourage their involvement in treatment decisions (6). Nevertheless, the considerable individual variation and nonlinear nature of functional recovery make precise prediction of ADL outcomes a persistent challenge (7).

In the field of stroke rehabilitation, numerous predictors of ADL recovery have been identified in previous studies, including demographic characteristics (e.g., sex and age), frailty status at admission (Clinical Frailty Scale [CFS] score), severity of neurological deficits (NIHSS score), and motor function assessments (8, 9). Nevertheless, their translation into routine clinical practice remains challenging. A key limitation is that most existing prediction models are derived from data collected during the acute phase of stroke, typically within neurology departments or stroke units in general hospitals (10, 11). Once patients transition beyond the acute phase and are admitted to rehabilitation wards for subacute interventions, rehabilitation physicians often lack timely and comprehensive access to these early-phase data, thereby reducing the feasibility and clinical utility of such models in guiding rehabilitation decisions. Furthermore, some early models are constrained by their reliance on specialized equipment (e.g., motor-evoked potentials) or by limited generalizability resulting from narrow study designs (9). Therefore, a significant clinical practice gap exists: rehabilitation physicians lack a dedicated prognostic tool designed for the subacute setting that leverages readily available, bedside clinical data collected upon a patient’s admission to predict future functional outcomes.

In response to this gap in clinical practice, we aim to develop and validate a clinical prediction model capable of accurately forecasting the ADL recovery level at 3 after rehabilitation intervention for subacute stroke patients (7–30 days post-stroke), based on routine bedside assessment data collected within 72 h of their transfer to rehabilitation wards. To achieve this, we utilize a large longitudinal cohort study with continuous recruitment over 4 years, employing the strengths of machine learning techniques for meticulous feature selection of multidimensional routine clinical data to identify the most critical predictors (7). On this basis, we construct and validate a robust logistic regression model, which will ultimately be converted into a user-friendly and intuitive nomogram (12). This tool will provide rehabilitation physicians with an efficient, evidence-based prognostic assessment tool that facilitates the development of personalized rehabilitation plans, advancing the precision of stroke rehabilitation decision-making.

## Methods

### Study design

The study was conducted in two Departments of Rehabilitation of Xiangyang Central Hospital, one in urban area of Xiangyang, and the other in suburban area of Xiangyang, Hubei, China. The Xiangyang Central Hospital is a tertiary hospital that provides intensive rehabilitation training by a multidisciplinary medical team to patients after emergency treatment. All patients underwent a structured assessment, and the patients’ rehabilitation needs were recorded by the physicians, physical therapists (PT), speech-language therapists (SLT) and nurses. The PT assessed the ADL. The physicians, SLT and nurses registered risk factors (e.g., sex, age, stroke stages, stroke sub-types, stroke areas).

### Participants

**Figure 1:**
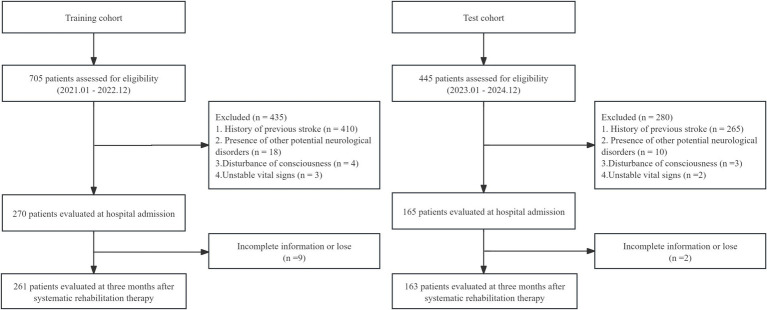
Flowchart of the research.

The data for this retrospective study were obtained from the electronic medical record system of Xiangyang Central Hospital. The study cohort comprised patients admitted to the hospital’s rehabilitation ward with a first-ever stroke between January 1, 2021, and December 31, 2024. Two independent cohorts were defined: a retrospective training cohort consisting of 270 patients enrolled between January 2021 and December 2022, and a validation cohort consisting of 165 patients enrolled between January 2023 and December 2024. Inclusion criteria were as follows: ischemic or hemorrhagic stroke confirmed by CT or MRI; first-ever onset; age ≥18 years; disease duration of 7–30 days; conscious and able to cooperate with assessment; and provision of written informed consent by the patient or their legal guardian. Exclusion criteria included: (1) the presence of other neurological diseases; (2) impaired consciousness; and (3) hemodynamic instability. The study was approved by the institutional ethics committee (No. 2024–098). Written informed consent was obtained from all participants in accordance with the Declaration of Helsinki. Reporting was conducted in accordance with the STROBE guidelines (Figure 1).

### Intervention and assessment

All patients received standardized inpatient rehabilitation after admission to the rehabilitation ward. The rehabilitation lasted for 3 months, during which patients underwent physical and occupational therapy five days per week. The specific content and intensity of therapy were individually tailored by the supervising therapist according to each patient’s functional status (13). The study was designed to develop a prediction model using clinical data obtained at admission. The primary outcome was the ADL independence status at 3 months after initiation of rehabilitation. ADL assessments were performed by PTs from the rehabilitation department at baseline (admission) and at 3-month follow-up, with data retrieved from electronic medical records.

### Data collection

Prior to study initiation, all staff received standardized training, including instruction on the use of the hospital’s electronic medical record system and data entry procedures. Data extraction and entry were independently conducted by two researchers, with a third researcher performing a random audit of 5% of the records to ensure accuracy and completeness. At admission, demographic characteristics were comprehensively documented, including age, sex, marital status, educational level, smoking history, drinking history, and primary source of medical expenses. Clinical data included systolic and diastolic blood pressure, stroke type (ischemic or hemorrhagic), stroke stage, BI score, Lindmark sitting and standing balance grades, VTE risk score, Braden score, fall/bed-fall risk score, spasticity assessment, EAT-10 swallowing function assessment, Holden walking function score, Brunnstrom hand function assessment, hemiplegic side, and disease duration. Medical history encompassed hypertension, hyperlipidemia, diabetes, coronary heart disease, family history, first-ever stroke, prior medical history, and stroke-related surgical history. Imaging data included the hemisphere involved and lesion location (cortex, subcortex, frontal, temporal, parietal, occipital, insular lobes, basal ganglia, thalamus, internal capsule, brainstem, and cerebellum).

### Outcome measure-ADL recovery

ADL recovery was assessed using the Barthel Index (BI), which ranges from 0 to 100. Baseline assessments were performed at admission. For model development, both primary and secondary outcomes were dichotomized as BI <60 (poor outcome, ADL dependence) or BI ≥60 (favorable outcome, ADL independence) (14, 15).

### Data analysis

#### Data preprocessing

The 3-month follow-up had a low attrition rate (9/270, 3.3% in the training cohort), with no significant differences in baseline characteristics observed between patients who completed follow-up and those lost to follow-up (all *p* > 0.05). Given the minimal proportion of missing outcome data, a complete-case analysis was performed for model development and validation, which is appropriate and unlikely to introduce meaningful selection bias under these conditions (16).

#### Feature selection and model development

A two-stage feature selection process was used to identify the most relevant predictors. First, univariate analyses were performed, and variables with *p* ≤ 0.05 were retained. Second, Elastic Net regression, combining L1 and L2 penalties, was applied to the retained variables. This method accounts for multicollinearity and selects a parsimonious predictor set by shrinking the coefficients of less informative variables. Multicollinearity among the final predictors was further assessed using the Variance Inflation Factor (VIF), with VIF < 5 indicating an acceptable level of correlation. The cohort was divided chronologically into a training set (January 2021–December 2022) and a validation set (January 2023–December 2024). A binary logistic regression model was then developed on the training set using the predictors selected by Elastic Net to estimate functional outcomes at 3 months.

#### Model performance and validation

Model performance was evaluated in both the training and validation cohorts using multiple metrics, including accuracy, the area under the receiver operating characteristic curve (AUC), sensitivity, specificity, positive predictive value (PPV), and negative predictive value (NPV). An AUC between 0.7 and 0.9 indicated acceptable discrimination, whereas an AUC > 0.9 indicated excellent discrimination. Internal validation was performed on the training set using 1,000 bootstrap resamples to assess overfitting and evaluate model stability.

#### Model interpretation

We used SHAP (SHapley Additive exPlanations) to interpret the contributions of individual predictors to model predictions. SHAP is an explainability framework based on Shapley values from cooperative game theory that fairly attributes the change in model output to each feature, providing both local (individual prediction) and global (overall importance) explanations. For the logistic regression model we computed SHA*p* values using the LinearExplainer implemented in the SHAP Python package; SHAP values were summarized across the cohort by mean absolute SHAP to rank global importance. Individual- and population-level effects were visualized using SHAP summary plots and force plots. Positive SHAP values indicate a contribution toward a higher predicted probability of a favorable outcome, whereas negative values indicate a contribution toward a lower predicted probability.

#### Nomogram construction and clinical utility assessment

To facilitate clinical application, the logistic regression model was translated into a nomogram. This graphical tool allows clinicians to calculate the probability of a favorable outcome by summing the points assigned to a patient’s individual characteristics. The nomogram’s calibration, reflecting the agreement between predicted and observed outcomes, was evaluated using calibration curves. Its clinical utility was further examined using decision curve analysis (DCA), which quantifies the net benefit of the model across a range of threshold probabilities.

### Statistical analysis

All statistical analyses were performed using Python (version 3.9.23) with the scipy.stats library. The normality of continuous variables was assessed using the Shapiro–Wilk test. Normally distributed variables were presented as mean ± standard deviation (SD) and compared using the independent t-test, whereas non-normally distributed variables were presented as median (interquartile range, IQR) and compared using the Mann–Whitney U test. Categorical variables were described as frequencies and percentages and compared using the χ^2^ test. A two-sided *p*-value of ≤ 0.05 was considered statistically significant.

## Results

### Participants’ characteristics

Of the 3,000 patients admitted to the hospital, 552 met the eligibility criteria for inclusion in the study. Of these, 424 patients completed 3-month follow-up assessments. Participants were further divided into a training cohort and a validation cohort according to their hospitalization periods, as illustrated in Figure 1. The training cohort consisted of 261 stroke patients who had 3-month follow-up data available, of whom 174 achieved a good ADL outcome (BI ≥60); their mean age was 62.85 ± 11.99 years. Demographic characteristics, clinical features, family history, and neuroimaging findings for all patients are summarized in Table 1.

**Table 1:** Demographic, clinical, medical history, and neuroimaging data of participants included in the study.

Characteristics	ADL dependent (*n* = 174)	ADL independent (*n* = 87)	*p-*value
Demographics			
Age, years, mean ± SD	62.85 ± 11.99	52.69 ± 13.10	0.01
Sex, *n* (%)			0.12
Male	114 (65.5%)	66 (75.9%)	
Female	60 (34.5%)	21 (24.1%)	
Marital status, *n* (%)			0.01
Married	164 (94.3%)	79 (90.8%)	
Unmarried or other	10 (5.8%)	8 (9.1%)	
Education level, *n* (%)			0.18
Primary school or below	45 (25.9%)	18 (20.7%)	
Junior high school	80 (46.0%)	33 (37.9%)	
Senior high school	30 (17.2%)	20 (23.0%)	
Junior college or above	19 (10.9%)	16 (18.4%)	
Smoking, *n* (%)	52 (29.9%)	30 (34.5%)	0.54
Drinking, *n* (%)	51 (29.3%)	29 (33.3%)	0.60
Health insurance type			0.91
Urban employee basic medical insurance	81 (46.6%)	38 (43.7%)	
Urban rural resident basic medical insurance	89 (51.1%)	47 (54.0%)	
Self-pay	4 (2.3%)	2 (2.3%)	
Medical history			
Hypertension, *n* (%)	154 (88.5%)	63 (72.4%)	0.01
Hyperlipidemia, *n* (%)	13 (7.5%)	6 (6.9%)	1.00
Coronary heart disease, *n* (%)	20 (11.5%)	11 (12.6%)	0.95
Diabetes, *n* (%)	38 (21.8%)	23 (26.4%)	0.50
Family history, *n* (%)	2 (1.1%)	2 (2.3%)	0.86
Clinical characteristics			0.43
Systolic blood pressure (mmHg)	128 (121, 140)	128 (120, 137)	0.42
Diastolic blood pressure (mmHg)	80 (75, 88)	79 (75, 87)	0.57
Disease course, day, median (IQR)	13 (9, 24)	10 (7, 17)	0.02
Stroke type, *n* (%)			0.32
Cerebral infarction	71 (40.8%)	44 (50.6%)	
Intracranial hemorrhage	96 (55.2%)	40 (46.0%)	
Subarachnoid hemorrhage	7 (4.0%)	3 (3.4%)	
Hemiplegic side, *n* (%)			0.001
No hemiplegia	1 (0.6%)	2 (2.3%)	
Left hemiplegia	62 (35.6%)	36 (41.4%)	
Right hemiplegia	66 (37.9%)	44 (50.6%)	
Bilateral paralysis	45 (25.9%)	5 (5.7%)	
Hemisphere of lesion, *n* (%)			0.89
No hemispheric lesion	42 (24.1%)	24 (27.6%)	
Left hemisphere	67 (38.5%)	34 (39.1%)	
Right hemisphere	53 (30.5%)	23 (26.4%)	
Bilateral hemispheres	12 (6.9%)	6 (6.9%)	
Sitting balance level, median (IQR)	0 (0.1)	2 (1.3)	0.001
Standing balance level, median (IQR)	0 (0.0)	0 (0.2)	0.001
VTE score, median (IQR)	3 (2.5)	1 (0.3)	0.001
BI score, median (IQR)	11 (0.25)	35 (20.51)	0.001
Braden score, median (IQR)	14 (12.16)	17 (15.18)	0.001
Fall/bed fall score, median (IQR)	7 (5.10)	6 (5.20)	0.35
Dysphagia, *n* (%)	125 (71.8%)	27 (31.0%)	0.001
Dystonia, *n* (%)	76 (43.7%)	25 (28.7%)	0.03
Gait dysfunction, *n* (%)	173 (99.4%)	86 (98.9%)	0.98
Hand function impairment, *n* (%)	172 (98.9%)	84 (96.6%)	0.38
Neuroimaging, *n* (%)			
Cortex	72 (41.4%)	28 (32.2%)	0.19
Frontal lobe	49 (28.2%)	13 (14.9%)	0.03
Temporal lobe	43 (24.7%)	13 (14.9%)	0.10
Parietal lobe	33 (19.0%)	12 (13.8%)	0.38
Occipital lobe	14 (8.0%)	10 (11.5%)	0.50
Insular lobe	6 (3.4%)	4 (4.6%)	0.97
Subcortical region	107 (61.5%)	50 (57.5%)	0.62
Basal ganglia	86 (49.4%)	43 (49.4%)	1.00
Brainstem	14 (8.0%)	13 (14.9%)	0.13
Internal capsule	0 (0%)	1 (1.1%)	0.72
Thalamus	15 (8.6%)	7 (8.0%)	0.92
Cerebellum	5 (2.9%)	4 (4.6%)	0.73

#### Feature selection

**Figure 2:**
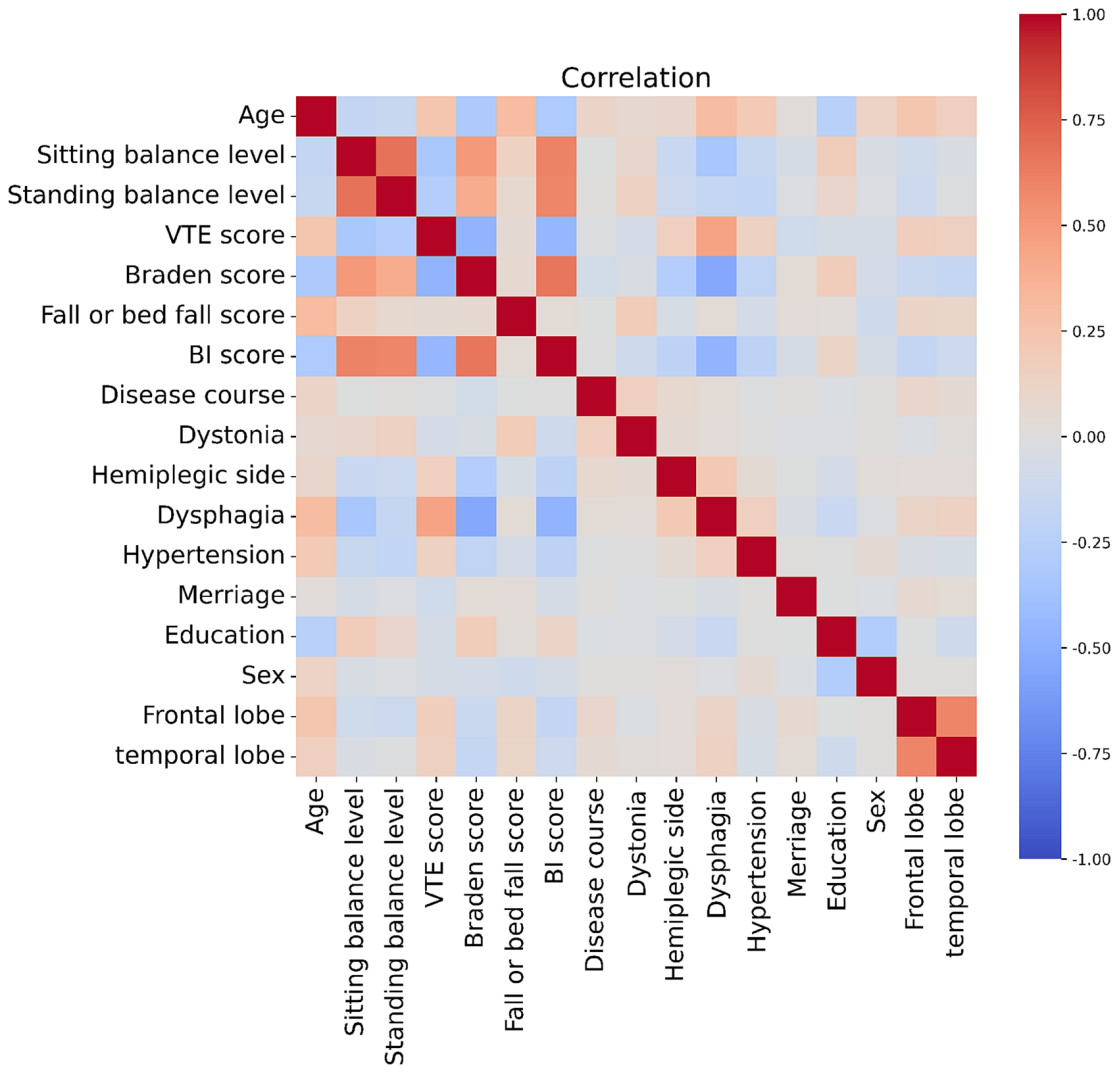
Correlation of selected features after univariate analysis for the 3-month outcome.

Univariate analyses were first conducted for all candidate variables, and those with *p* ≤ 0.05 were retained. Subsequently, correlation analysis was performed to assess the associations among these selected variables; results are presented in Figure 2. The variance inflation factor (VIF) for all retained variables was <5, indicating no significant multicollinearity (Supplementary Figure S1). Based on the selected features, Elastic Net regression was applied, with tenfold cross-validation on the training set used to determine optimal parameters. Model performance across different values of l1_ratio and regularization strength is shown in Supplementary Figure S2. After identifying the optimal l1_ratio, the trajectories of feature coefficients as a function of the L1 norm are displayed in Supplementary Figure S3. As regularization strength increased, cross-validated AUC values first rose and then decreased (Supplementary Figure S4); notably, even when the model was reduced to a single feature, the mean AUC for predicting 3-month ADL independence approached 0.8. Given minimal differences in performance when models with different numbers of retained features were evaluated on the training set, features were ranked based on the order in which their coefficients were reduced to zero during Elastic Net regularization. For 3-month ADL independence, the most influential predictors in descending order were: Braden score, BI score, age, sitting balance grade, presence of oral dysfunction, presence of spasticity, standing balance grade, VTE score, hypertension, disease duration, hemiplegic side, frontal lobe involvement, and marital status.

#### Model development and performance comparison

**Figure 3:**
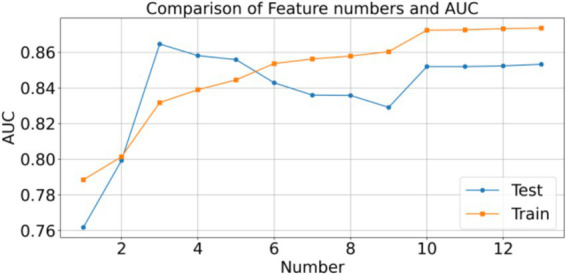
Comparison of AUC values for prediction models constructed with different numbers of features in the training and validation sets.

Based on the feature ranking obtained in the previous section, different numbers of features were retained, and logistic regression (LR) was used to build predictive models on the training set. The comparison of AUC between the training and validation sets is shown in Figure 3. For predicting 3-month ADL independence, when the number of features was 3, the validation set AUC reached its maximum value (approximately 0.87); therefore, three features were selected to construct the nomogram. The final predictors for 3-month ADL independence were Braden score, BI score, and age. The performance of the final prediction model in the training and validation sets is presented in Supplementary Table S1.

#### Model explanation

**Figure 4:**
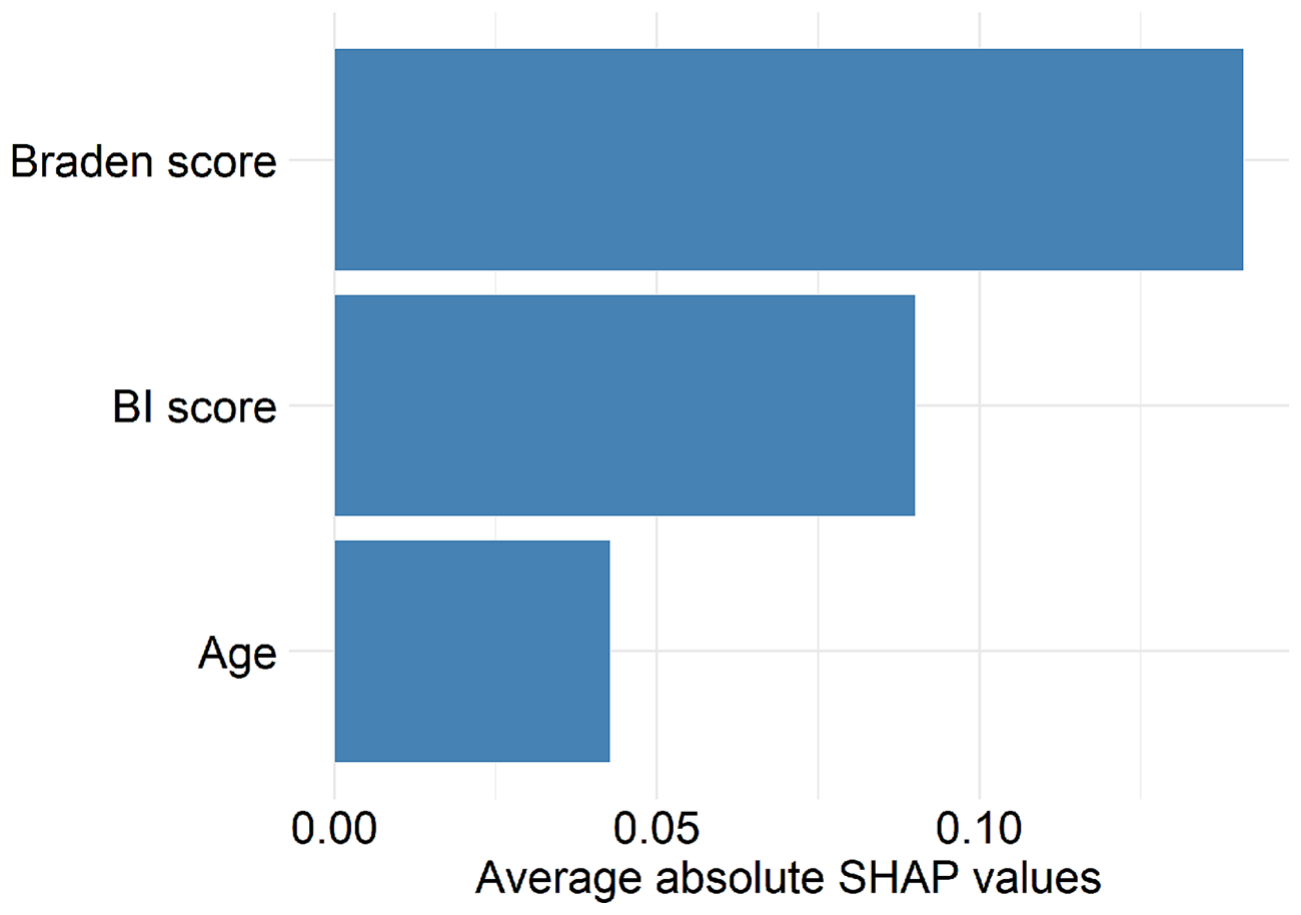
Feature importance based on SHAP analysis for the 3-month prediction model.

We computed SHAP values to interpret predictor contributions for the logistic regression model using the SHAP Python package, calculating SHAP only for the three features retained for model construction. For the linear model we used LinearExplainer to compute per-feature SHAP values. SHAP values were summarized across the cohort by the mean absolute SHAP to rank global feature importance. The SHAP analysis revealed that the Braden score was the most significant predictor for the 3-month outcome, followed by BI score and age (Figure 4).

#### Development and validation of the nomogram model

**Figure 5:**
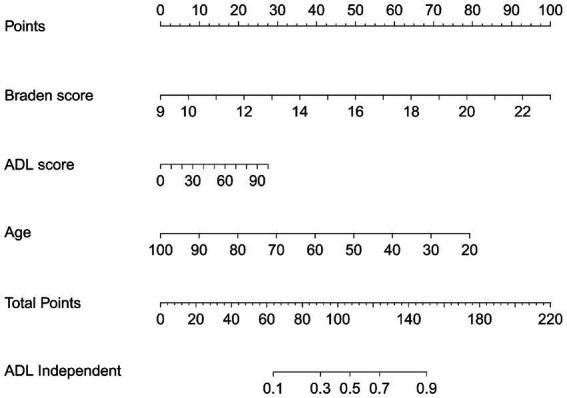
Nomogram for predicting ADL recovery at 3 months in stroke patients (ADL-3 M). Point allocation was based on baseline Braden score, BI score, and age. To use the nomogram, first locate the patient’s value on each predictor axis and draw a vertical line down to the ‘Points’ scale to determine the score for each variable. The individual points are then summed to obtain the ‘Total Points,’ which is projected downward to the ‘ADL independence’ scale to estimate the probability of achieving ADL independence.

**Figure 6:**
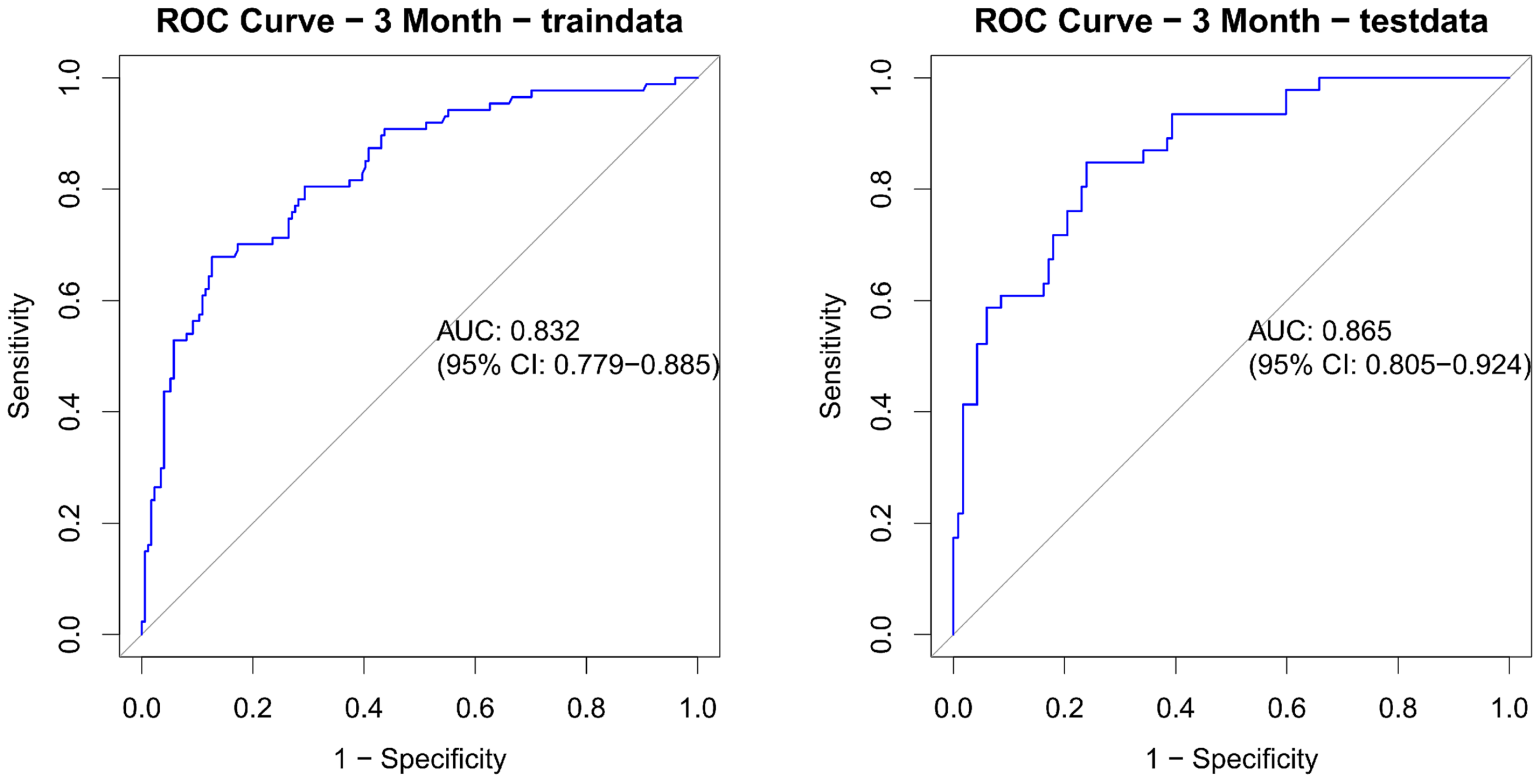
ROC curves of the ADL-3 M nomogram model in the training and validation cohorts.

**Figure 7:**
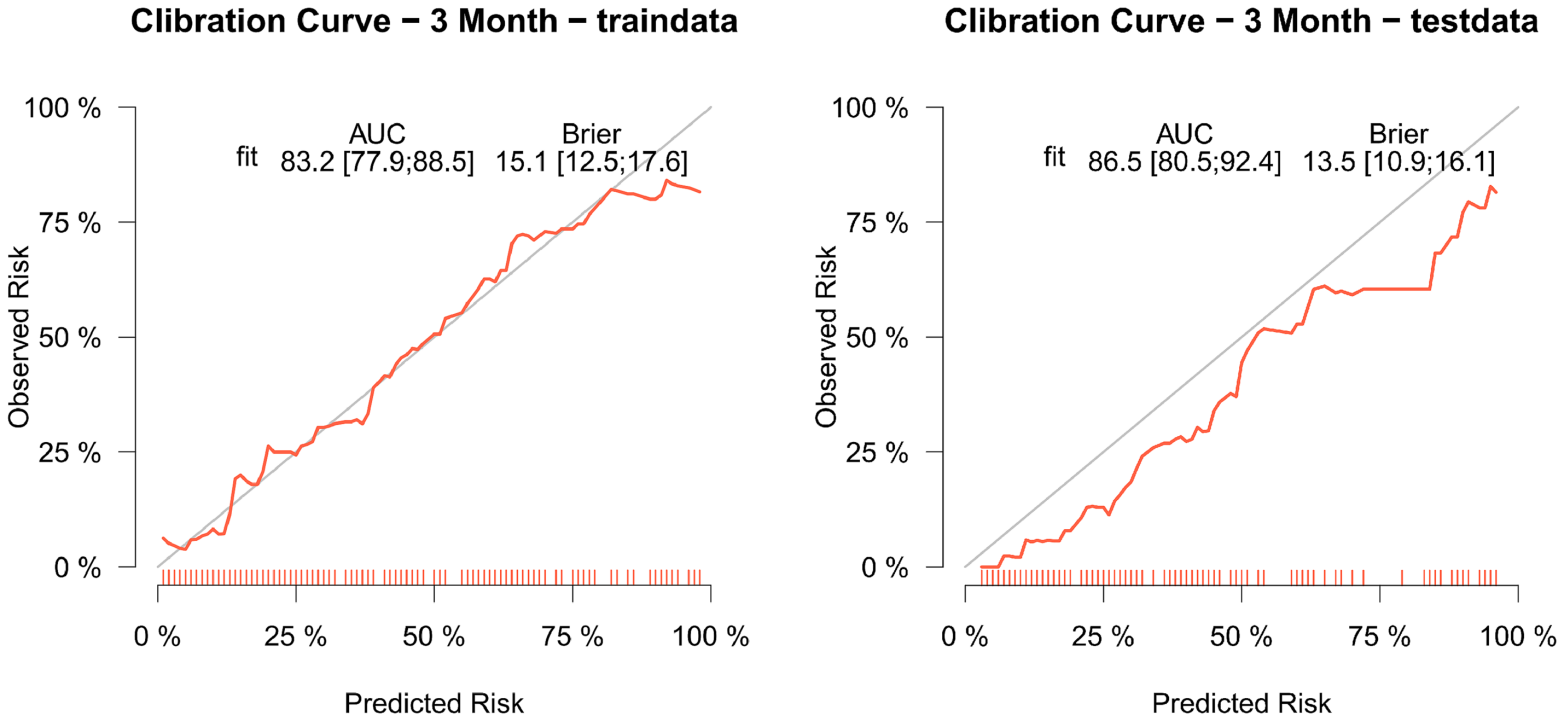
Calibration curves of the ADL-3 M nomogram model in the training and validation cohorts.

**Figure 8:**
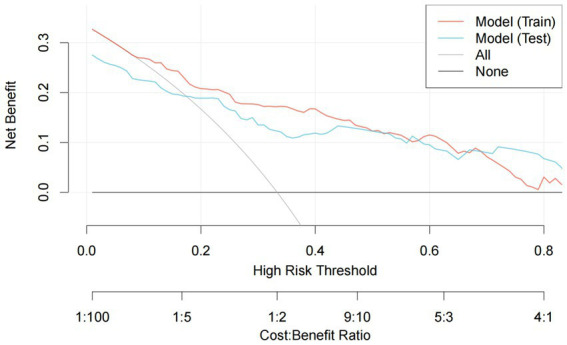
Decision curve analysis of the ADL-3 M nomogram model in the training and validation cohorts.

A nomogram model based on three key clinical features was developed to predict ADL independence at 3-month follow-up (Figure 5). The ADL-3 M model incorporated Braden score, BI score, and age. In the training cohort, the model achieved an AUC of 0.832 (95% CI: 0.779–0.885). In the validation cohort, the model achieved an AUC of 0.866 (95% CI: 0.806–0.926) (Figure 6). Calibration curves demonstrated strong concordance between predicted and observed outcomes (Figure 7). Decision curve analysis (DCA) further demonstrated that application of the nomogram provided a clear clinical net benefit across a wide range of threshold probabilities (Figure 8).

## Discussion

Accurate prediction of ADL recovery during the early intervention phase in stroke patients is essential for optimizing rehabilitation strategies. Many existing prediction models rely on acute-phase data, which are often difficult for rehabilitation physicians to obtain when planning treatment, thus limiting their applicability in rehabilitation settings. This study, based on real-world clinical data, utilized machine learning feature selection techniques to identify the most critical predictors affecting outcomes from a range of variables (17, 18). Based on this, we developed and validated an efficient logistic regression model (ADL-3 M), which was then converted into an intuitive nomogram (19).

Specifically, the ADL-3 M model, which only requires age, admission BI score, and Braden score, effectively predicts functional outcomes at 3 months. The study confirms that, during the critical subacute phase, three easily accessible indicators can provide reliable evidence-based references for ADL recovery prediction, offering a practical and user-friendly quantitative prognostic tool for clinical application.

Regarding model performance, the ADL-3 M model demonstrated good overall discrimination in both training and validation cohorts (training AUC: 0.832; validation AUC: 0.866). A key feature of the model is its high specificity (86% in the validation cohort). From a clinical perspective, high specificity means the model can reliably identify patients who are likely to remain ADL-dependent at 3 months. This characteristic is vital for rehabilitation practice, as it provides an objective foundation for early physician-patient discussions, helps set realistic rehabilitation goals, and enables the prioritization of limited rehabilitation resources for the highest-risk patients. However, the model’s sensitivity was moderate (61%), indicating that it performs moderately well in identifying patients who will achieve ADL independence. This may be influenced by unaccounted factors, such as the patient’s neuroplastic recovery potential, rehabilitation adherence, psychological state, and social support (20–23).

The ADL-3 M model showed a high Negative Predictive Value (NPV) of 85%, which is one of the most clinically valuable findings of this study. A high NPV means that when the model predicts a patient will remain ADL-dependent, this prediction is highly reliable. In clinical practice, this feature acts as a reliable ‘warning tool,’ accurately identifying high-risk individuals who require long-term, intensive rehabilitation resources (24). As a retrospective cohort study, this research faces the common issue of loss to follow-up. However, the high NPV ensures that patients with poor prognoses, who most need ongoing medical attention, are not overlooked by the clinical management system due to loss to follow-up, thereby ensuring continuity of care (25).

In terms of variable importance, the key predictors in the ADL-3 M model were Braden score, BI score, and age. Our findings align with previous studies, which indicate that the BI score at admission is strongly associated with long-term ADL recovery (26). It serves as a functional baseline when patients enter the subacute rehabilitation phase, reflecting both the initial severity of stroke-induced neurological damage and the patient’s remaining functional capacity (27). A higher baseline score often suggests better motor, sensory, and cognitive reserves, which are crucial for subsequent rehabilitation efforts.

Age is a well-established predictor of post-stroke prognosis (28). Older individuals generally have poorer ADL recovery outcomes, likely due to the combined effects of factors such as reduced physiological reserves, increased comorbidities, diminished postural adjustment ability, and inefficient muscle recruitment, all of which further hinder their ability to perform functional activities (29, 30).

An important finding of this study is the high predictive value of the Braden score. Traditionally used for assessing pressure ulcer risk, the Braden scale evaluates various dimensions, including mobility, sensory perception, nutrition, moisture, and activity. A low Braden score serves as a comprehensive indicator reflecting the patient’s overall frailty, immobility, and nutritional status (8, 31). Subscales such as activity and mobility are directly related to ADL performance, while others, such as nutrition, influence neurorepair, muscle recovery, and rehabilitation tolerance. The Braden score can be understood as an easily accessible and highly integrated surrogate variable (32), and its predictive value for ADL recovery arises from its broad reflection of multiple key physiological factors that affect rehabilitation potential.

### Study limitations

First, the sample size in this study is limited. Despite employing various methodological strategies to enhance model robustness, such as feature selection to reduce model complexity and performing validation on an independent chronological cohort, the inherent limitations of a small sample size may still impact the model’s stability and predictive accuracy. In healthcare, creating large-scale, high-quality disease-specific cohort data is a significant challenge (7). Second, the range of predictive variables is limited by the availability of data from electronic medical records. Some important predictors that have been identified in the literature as relevant to stroke prognosis, such as cognitive function and psychological state, were not included in this study (33). Future prospective studies should aim to gather more detailed data to develop more comprehensive predictive models (32). Finally, the data-driven nature of machine learning models inherently limits their generalizability. The model developed in this study performs well on the specific dataset used, but its performance may degrade when applied to other medical centers, different regions, or patient populations with different baseline characteristics. A more constructive recommendation is for healthcare institutions to use the findings from this study—particularly the core predictors identified—as a reference to calibrate, optimize, or develop localized predictive models suited to their specific clinical contexts.

## Conclusion

This study successfully developed and validated a prediction model (ADL-3 M) based on routine clinical data from the subacute phase, effectively addressing the gap in stroke rehabilitation prognostic tools during this critical stage. The study demonstrated that three easily accessible bedside indicators—age, admission BI score, and Braden score—can reliably predict a patient’s ADL independence at 3 months post-rehabilitation. The ADL-3 M model, when converted into a nomogram, offers an objective and quantitative tool for prognostic evaluation. With its high specificity and negative predictive value, this model can accurately identify high-risk patients with poor outcomes, providing significant clinical value for developing personalized rehabilitation plans, optimizing medical resource allocation, and fostering effective doctor-patient communication.

## Data Availability

The raw data supporting the conclusions of this article will be made available by the authors, without undue reservation.
