# Supplementary material for: Development and validation of a nomogram for predicting ADL outcomes in patients undergoing subacute stroke rehabilitation based on machine learning and standard bedside clinical data: a retrospective cohort study
Source: Front Neurol. 2026 Jun 19;17:1831565. doi: 10.3389/fneur.2026.1831565 (PMC13328032; doi:10.3389/fneur.2026.1831565)
Supplement: Supplementary file 1 [file Supplementary_file_1.DOCX]

**
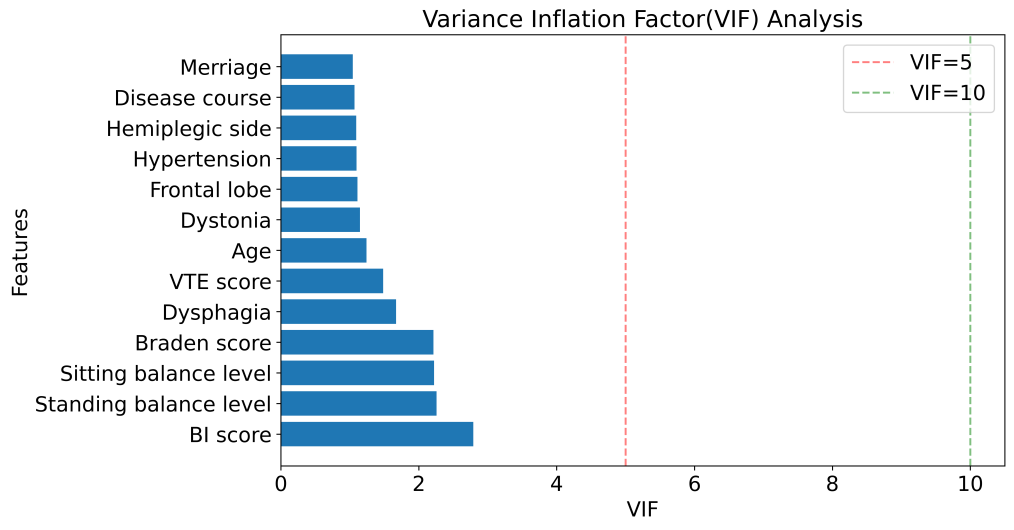
**

**Figure S1**. Collinearity analysis of features retained after univariate analysis in the training cohort. (Left) 3-month outcomes; (Right) 6-month outcomes.


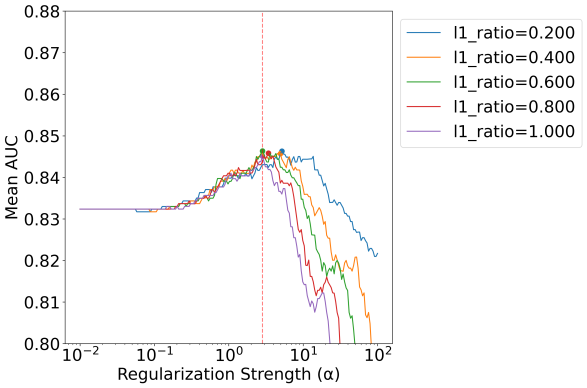


**Figure S2**. Model performance across varying regularization strengths under different L1 ratios (l1_ratio).


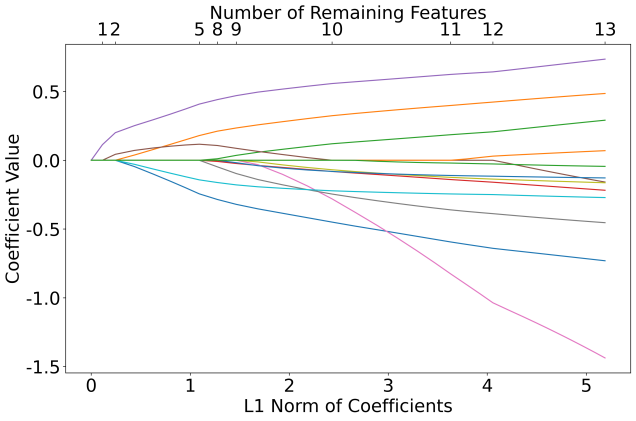


**Figure S3**. Changes in feature coefficients with varying regularization strengths.


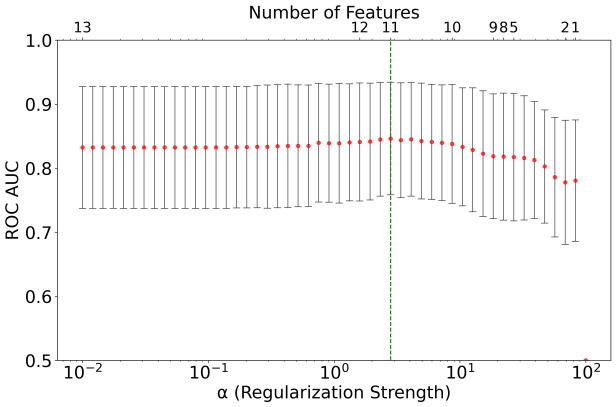


**Figure S4**. Changes in AUC with varying regularization strengths in the training set.

**Table S1**. Performance of the optimal feature selection models in the training and validation cohorts.

| Follow-up |  | Sensitivity | Specificity | ACC | PPV | NPV | AUC |
| --- | --- | --- | --- | --- | --- | --- | --- |
| 3 months | validation | 0.609 | 0.855 | 0.785 | 0.622 | 0.847 | 0.866 |
|  | training | 0.574 | 0.897 | 0.789 | 0.735 | 0.808 | 0.832 |
|  | training | 0.656 | 0.897 | 0.808 | 0.788 | 0.818 | 0.853 |

ACC = Accuracy; PPV = Positive Predictive Value; NPV = Negative Predictive Value; AUC = Area Under the ROC Curve.
